# Pharmacological Manipulation of Early Zebrafish Skeletal Development Shows an Important Role for Smad9 in Control of Skeletal Progenitor Populations

**DOI:** 10.3390/biom11020277

**Published:** 2021-02-13

**Authors:** Georgina L. K. McDonald, Mengdi Wang, Chrissy L. Hammond, Dylan J. M. Bergen

**Affiliations:** 1 School of Physiology, Pharmacology and Neuroscience, Faculty of Life Sciences, University of Bristol, Bristol BS8 1TD, UK; gm15811@bristol.ac.uk (G.L.K.M.); mengdi.wang@bristol.ac.uk (M.W.); 2 Musculoskeletal Research Unit, Translational Health Sciences, Bristol Medical School, University of Bristol, Bristol BS10 5FN, UK

**Keywords:** osteoblast, biomolecule, musculoskeletal, zebrafish, BMP-signalling, Smad9, osteoporosis

## Abstract

Osteoporosis and other conditions associated with low bone density or quality are highly prevalent, are increasing as the population ages and with increased glucocorticoid use to treat conditions with elevated inflammation. There is an unmet need for therapeutics which can target skeletal precursors to induce osteoblast differentiation and osteogenesis. Genes associated with high bone mass represent interesting targets for manipulation, as they could offer ways to increase bone density. A damaging mutation in *SMAD9* has recently been associated with high bone mass. Here we show that Smad9 labels groups of osteochondral precursor cells, which are not labelled by the other Regulatory Smads: Smad1 or Smad5. We show that Smad9^+^ cells are proliferative, and that the Smad9^+^ pocket expands following osteoblast ablation which induced osteoblast regeneration. We further show that treatment with retinoic acid, prednisolone, and dorsomorphin all alter Smad9 expression, consistent with the effects of these drugs on the skeletal system. Taken together these results demonstrate that Smad9^+^ cells represent an undifferentiated osteochondral precursor population, which can be manipulated by commonly used skeletal drugs. We conclude that Smad9 represents a target for future osteoanabolic therapies.

## 1. Introduction

Bone is a mineralised tissue, in which dynamic remodelling is tightly regulated by the activity of osteoclasts (which resorb matrix) and osteoblasts and osteocytes (which deposit bone matrix). This tight coupling of bone resorption and deposition is essential to maintain a healthy skeleton capable of functioning efficiently. When this balance is perturbed it can lead to various bone remodelling related diseases such as osteopetrosis (excessive bone strength) or osteoporosis (OP; reduced bone strength) [1]. OP is a common age-related bone degeneration disease that is characterised by a low bone mineral density (BMD) and reduced bone mass resulting in increased bone fragility and fracture incidence. In the European Union (EU), over 27.6 million people over the age of 50 suffer from OP [2]. Treating these fragility fractures cost the EU €37 billion in 2010 and this is predicted to increase to €46.5 billion by 2025 due to our aging population, with only a small percentage of the cost representing pharmacological intervention [3]. Therefore, there is scope to fulfill a large demand to develop drug treatments to lower the incidence of fragility fractures. Currently antiresorptive drugs, such as bisphosphonates which block the activity of osteoclasts and therefore bone degradation, are the mainstay of OP treatment. However, there are side effects associated with long term use, including hip fractures, gastrointestinal discomfort, and osteonecrosis in the jaw. There are few affordable and minimally invasive treatments aimed at enhancing bone formation (osteoanabolic). Teriparatide is a costly daily subcutaneously administered drug which consists of recombinant human parathyroid hormone (PTH) [4]. However, it cannot be used long term as its strong osteoanabolic effect increases the risk of osteosarcomas [5]. Therefore, there is a demand in the field to discover new osteoanabolic drug targets which are minimally invasive treatments that could restore the balance of bone degradation and formation in OP patients.

New osteoanabolic drug targets have previously been discovered by studying individuals with high bone mass (HBM). HBM is a rare genetic disorder which results in increased bone mass and BMD providing resistance to fracture [6]. A striking example was the discovery that mutations in sclerostin (*SOST*) caused HBM in several pedigrees [7,8]. As *SOST* is predominantly expressed in osteocytes to block canonical WNT signalling, this prompted the development of Romosozumab antibody treatment, which blocks SOST leading to stimulation of the canonical WNT signalling pathway. Romosozumab is now used to enhance bone formation in OP patients [7]. By using a similar human genetic approach of genome-wide association studies (GWAS) and whole-genome/exome sequencing (WGS/WES) *SMAD9* was identified as a novel osteoanabolic candidate, this could therefore make SMAD9, like SOST, a potential anabolic drug target for OP [9].

The monogenic HBM mutation caused a p.Leu22Pro substitution that is predicted to disrupt the MAD-homology (MH) 1 DNA binding domain of SMAD9 (previously known as SMAD8 or MADH9). SMAD9 is a co-transcription factor with SMAD1 and SMAD5, called regulatory SMADs (R-SMADs), in the SMAD dependent Bone Morphogenetic Protein (BMP) signalling pathway. The other R-SMADs, SMAD2 and SMAD3, are BMP independent and are part of the TGF-β pathway [10]. In canonical BMP signalling, binding of BMP ligands to type I and II BMP receptors (BMPR) dimerise leads to a conformational change of the intracellular domain of the BMPR-II. This allows its kinase domain to phosphorylate R-SMADs at the C-terminal SSXS motif and trimerisation of SMAD1, SMAD5, and SMAD9 to occur. It then forms a tetrameric complex with SMAD4 and traffics from the cytosol to the nucleus to regulate transcription of target genes. Within this complex, SMAD9 has the unique capacity to repress transcription whereas SMAD1 and SMAD5 activate transcription [11]. The R-SMADS are composed of two MH domains of which the N-terminal MH domain bind to DNA whereas the MH2 domain can bind co-factors and allows the oligomerisation to occur. The BMP signalling pathway, and BMPR function have previously been pharmacologically targeted. For example, dorsomorphin is a BMPR antagonist which prevents the occurrence of a conformational change leading to inadequate phosphorylation of R-SMADS [12]. As SMAD9 is unique in inhibiting transcription in the R-SMAD complex, and has been identified as a HBM gene, it appears to be an attractive target gene. However, it is important to first assess the suitability of SMAD9 as a target by characterising spatiotemporal activity in vivo following manipulation of the skeletal system, in order to identify which cell types would respond upon its manipulation in vivo.

In recent years there has been a surge in the experimental use of zebrafish and medaka as vertebrate models for disease and drug discovery. The release of the zebrafish genome in 2013 showed that zebrafish physiology is highly conserved with humans; they share 70% of all genes and 85% of disease genes [13]. Zebrafish have a similar skeletal physiology to humans, they undergo both endochondral and intramembranous ossification and they form synovial joints within the jaw [14,15]. The development of the zebrafish craniofacial skeleton is rapid [16], and unlike rodent models, development of bones can be dynamically visualised in zebrafish larvae as early as 3 days post fertilisation (dpf). This fast development of complete organ systems has resulted in multiple small-molecule drug screens in zebrafish for a range of diseases [17]. Compounds can be easily administered in multi-well plates containing the fish and media allowing for drug screening. This in combination with several established reporter lines relevant to musculoskeletal biology and its signalling pathways make it an attractive model for drug discovery [18]. In addition to reporter lines, there are conditional transgenic lines allowing chemical ablation of osteoblasts which permit to study the effects of pharmacological manipulation on skeletogenic precursors pools during bone regeneration [19]. One additional advantage of manipulating early skeletogenesis in larval zebrafish is that effects on bone made by osteoblasts can be separated from those of osteoclasts, since osteoclasts are not fully formed in zebrafish until around 10 days of development [20,21].

More specifically, zebrafish larvae have been used to quickly screen for a range of bone metabolic drug treatments due to the ease of bone mineralisation staining and quantification [22]. Drugs screened in zebrafish have already made it to the clinic, such as BMP type-1 receptor (BMP1R) antagonist dorsomorphin which was discovered in an early embryogenesis phenotype screen and it is now used to treat lymphoma [23,24]. A number of other biomolecules, including clinically relevant drugs such as prednisolone and alendronate have been used to give insight into control of cell behaviour during bone development [25] homeostasis [26] and regeneration [27] in the zebrafish and in ex vivo cultures of the bony scales [28,29].

Here we describe in detail the expression of Smad9 during musculoskeletal development in larval zebrafish. We show that Smad9 is expressed in a population of osteochondral precursors, which are capable of proliferation and contribute to regeneration of osteoblasts following their ablation. We further demonstrate that the population of Smad9 expressing cells can be manipulated by exposure to a number of biomolecules known to affect the skeleton. Taken together, we suggest that Smad9 is both a useful biomarker for skeletal precursor populations and a suitable target for follow-up therapeutic manipulation of skeletogenesis.

## 2. Materials and Methods

### 2.1. Zebrafish Husbandry and Transgenic Lines

Zebrafish (*Danio rerio*) in AB/TL background were maintained under normal husbandry conditions [30]. Experiments were locally ethically reviewed (AWERB at University of Bristol, Bristol, UK) and performed under UK Home Office project license (30/3804. Transgenic lines have been previously described; *Tg(Ola.sp7:NLS-GFP)* (referred to as *sp7:GFP*) [31], *Tg(osterix:mCherry-NTRo)^pd46^* (referred to as *sp7:mCherry-NTR*) [19], *Tg(5xBMPRE-Xla.Id3:GFP)* (referred to as *BMPre:GFP*) [32], *Tg(-4.9sox10:egfp)^ba2^* (referred to as *sox10:GFP*) [33], *Tg(7xTCF.XlaSiam:nlsGFP)* (referred to as *Wnt:GFP*) [34] and *Tg(fms:GFP)^SH377^* (referred to as *cfms:GFP*) [35]. Larvae were maintained in 30% Danieau’s solution (Stock: NaCl 1740 mM, KCl 21 mM, MgSO_4_ 12 mM, Ca(NO_3_)_2_ 18 mM and HEPES buffer 150 mM in 1 L distilled H_2_O).

### 2.2. Pharmacological Treatment

Retinoic acid (RA), dorsomorphin (DM), and prednisolone (PD) (all from Sigma-Aldrich, St Louis, MI, USA) were dissolved in dimethylsulfoxide (DMSO) to a working stock concentration of 10 mM, 40 mM, or 100 mM, respectively. Zebrafish embryos (3 dpf) incubated with Danieau’s solution were randomly divided into four groups: 0.01% DMSO, 1 μM RA, 4 μM DM and 25 μM PD. These concentrations were used based on previous publications and were further confirmed after titration by quantifying the RA effect on operculum length (treated from 3 dpf to 5 dpf at 0.5 μM, 1 μM, and 2 μM), DM effect on embryonic dorsalisation after gastrulation (treated from 4 hpf to 24 hpf at 2 μM, 4 μM, 8 μM, and 16 μM), and PD effect on immune cell number (treated from 3 dpf to 5 dpf at 12.5 μM, 25 μM, and 50 μM) against 0.1% DMSO control (Figure S1) [12,36,37]. Each group with the same number of embryos, 8 per group, were ablated with 5 μM nifurpirinol (NFP, Sigma-Aldrich, 32439) for 6 h firstly, then treated with related drugs for 48 h (from 3.25 dpf onward), refreshing the solution twice a day, 0.01% DMSO, 1 μM RA, 4 μM DM and 25 μM PD, from 3.25 dpf to 5.25 dpf. At 5.25 dpf larvae were lethally anesthetised with tricaine (MS222) and fixed as described below.

### 2.3. Immunohistochemistry

Developmentally staged larvae (after euthanisation in MS222) were fixed in 4% paraformaldehyde (1 h), dehydrated to 100% methanol, and stored at −20 °C before staining. Immunolabeling was done as previously described [21]. Primary antibodies were anti-Smad9 (ab96698, Abcam, Cambridge, MA, USA) used at a 1:200 dilution, anti-Col2a1 (II-II6B3, DSHB, University of Iowa, Iowa, IA, USA) used at 1:20 and anti-GFP (chicken polyclonal, Abcam, ab13970) used at a 1:300 dilution in blocking buffer (5% horse serum). Additionally, rabbit anti-Smad1 (1:100 dilution, sc-6031-R, Santa Cruz, Dallas, TX, USA), rabbit anti-Smad5 (1:200 dilution, ab227090, Abcam), and rat anti-mCherry [16D7] (1:100 dilution, M11217, Invitrogen, Carlsbad, CA, USA). Primary antibodies were used in 5% horse serum in 0.2% PBS-Triton-X100 blocking buffer. Secondary antibodies Alexa Fluor 488 anti-rabbit, Alexa Fluor 568 anti-chicken, Alexa Fluor 647 anti-mouse (A21206, A11041, and A31571 respectively, Invitrogen, Carlsbad, CA, USA), and Dylight 650 anti-Rat (SA5-10029, ThermoFisher, Waltham, MA, USA) were used at a 1:500 dilution. Samples were mounted in 1% low melting point agarose and imaged as described below.

### 2.4. RNAscope In Situ Hybridisation

In situ hybridisation (ISH) was performed by using RNAscope ISH Technology (ACD Bio) according to the manufacturer’s instructions. In brief, 5 dpf larvae were fixed in 4% PFA for 2 h and dehydrated in methanol before digestion in Protease Plus for 30 min. The *runx2a* probe (1:50) was hybridised at 40 °C overnight followed by a second fixation for 10 min. The probe channel was then amplified and developed before imaging.

### 2.5. Proliferation Assay

Proliferation of the larvae was measured using the Click-iT EdU imaging kit (Invitrogen) according to the manufacturer’s instructions. In brief, larvae were treated with 400 μM EdU in Danieau’s solution for 8 h on 4 dpf. They were fixed in 4% PFA and then incubated in the Click-iT reaction cocktail for 30 min.

### 2.6. Alcian Blue Staining

Alcian blue 8 GX (Sigma-Aldrich) was used to visualise cartilage glycosaminoglycans (GAGs). Larvae (5 dpf) were fixed in phosphate buffered formaldehyde (3.5% formaldehyde diluted in 100 mM monosodium phosphate buffer supplemented with 25 mM disodium phosphate) for an hour. Following fixation, larvae were dehydrated to 80% ethanol (EtOH). Larvae were incubated in freshly made Alcian blue staining solution (0.02% Alcian blue in 70% ethanol supplemented with 80 mM MgCl_2_) at room temperature for 1 h. Larvae were washed in 50% EtOH, and subsequently washed in deionised water with 0.2% Triton-X100. Pigmentation was bleached in a solution of 10% KOH, 1.5% H_2_O_2_, in deionised H_2_O) which was followed by a wash in 10% KOH, 10% Glycerol, and cleared in 70% glycerol, prior to imaging (ventral and lateral views) with a Leica stereomicroscope.

### 2.7. Image Acquisition and Processing

Live larvae were imaged at 4 dpf and 5 dpf and were anesthetised in 0.1 mg mL^−1^ MS222 under a fluorescent stereomicroscope (Leica). For Lightsheet fluorescence microscopy (Zeiss Z.1), 3 dpf larvae were embedded in 1% low-melting point agarose and were imaged for up to 6 h with an acquisition frequency of 15 min. Immunohistochemistry and ISH samples (n ≥ 4) were imaged using a confocal laser scanning microscopy (Leica, Buffalo Grove, IL, USA, SP5II AOBS attached to a Leica DM I6000 inverted epifluorescence microscope) using a 10× PL APO CS, 20× HC PL APO CORR or 40× PL APO CS (4.0, 0.7 and 1.3 numerical aperture respectively) lenses. The larvae were mounted laterally or ventrally in 1% low melting point agarose (dissolved in Danieau’s solution) before imaging. Images were processed using Fiji [38] and linear brightness and contrast adjustments were applied. All IHC and ISH images are representative full projection images of confocal stacks. Three-dimensional (3D) volume renders were generated in Imaris software package (Oxford Instruments) of the operculum and Smad9^+^ population of cells at the dorsal tip of the operculum. Thresholds were maintained between 40–60% and the volume function was used to measure the population of Smad9^+^ cells.

### 2.8. Protein Sequence Alignment

Human (ENSP00000369154), mouse (ENSMUSP00000029371), and zebrafish (ENSDARP00000117800) Smad9 protein sequences were downloaded from ENSEMBL (release 99) and aligned with Clustal Omega (v1.2.4) algorithm to identify sequence conservation [39].

### 2.9. Statistics

Statistical analyses were performed using GraphPad Prism 8.00 (GraphPad Software, San Diego, CA, USA). One-way ANOVA with Tukey’s multiple comparisons test and unpaired *t*-tests were performed where appropriate. Standard error of the mean is displayed on each dot plot with *n* ≥ 4 in all conditions. The statistical significance system used is as follows: ^ns^ *p* > 0.05, * *p* ≤ 0.05, ** *p* ≤ 0.01, *** *p* ≤ 0.001. **** *p* ≤ 0.0001. Sample sizes are indicated in the figure legends, and significant P values are shown on the figures. All tests met standard assumptions, and the variation between each group is shown. Sample sizes were chosen based on previous, similar experimental outcomes and were based on standard assumptions. No samples were excluded. Larvae were randomly allocated to treatment conditions. A subset of the data was analysed by a researcher blinded to the treatment.

## 3. Results

### 3.1. Smad9 Is Highly Expressed in Putative Osteochondral Progenitors during Embryonic Development

**Figure 1:**
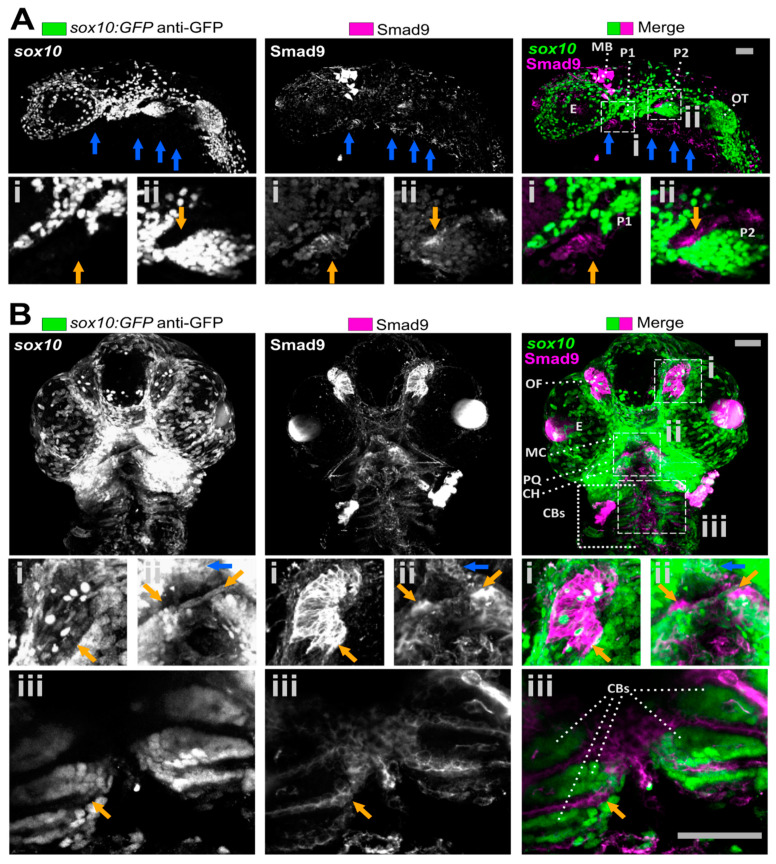
Smad9 is expressed adjacent to sox10 positive osteochondral progenitors in early zebrafish development. (**A**) Lateral view of a 24 hpf *sox10:GFP* transgenic embryo showing Smad9 expression ventrally (blue arrows) and near cranial neural crest pharyngeal pouches (orange arrows in insets). Orange arrows in the insets show Smad9 expression surrounding the anterior two pharyngeal pouches which will give rise to the MC and PQ (P1), and CH (P2). Note that Smad9 is also expressed at a low level in most GFP positive cells. (**B**) Ventral view of 48 hpf *sox10:GFP* transgenic fish showing Smad9 expression surrounding GFP positive cells. Insets show high Smad9 expression in the olfactory bulb (i, orange arrow), at future MC (blue arrow) and ceratohyal (orange arrow) joint symphyses (ii), and surrounding the CBs (iii, orange arrow). CBs, ceratobranchial arches; CH, ceratohyal; E, eye; MC, Meckel’s cartilage; OF olfactory placode; OT, otolith; P, pharyngeal pouch; PQ, palatoquadrate MB, midbrain. Scale bar is 50 µm in all images.

**Figure 2:**
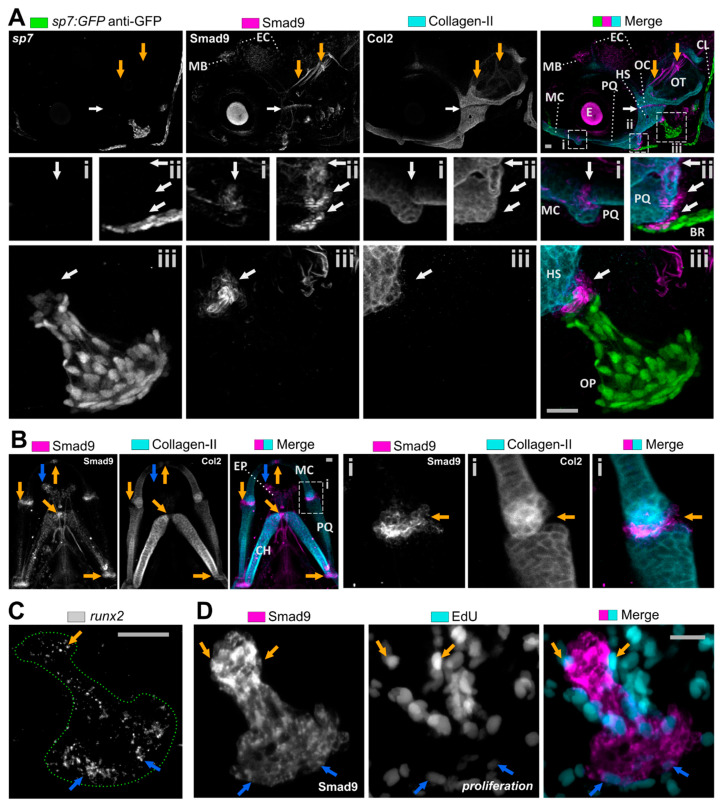
Smad9 is expressed in cell populations marking joint symphyses and interfaces of cartilage and bone of the craniofacial skeleton. (**A**) Lateral view of 4 dpf individual showing Smad9 expression in the craniofacial skeleton in cell populations located at joint symphyses of otic cartilage (OC) and PQ (white arrow) and PQ and MC (inset i), and at cartilage and bone interfaces (inset ii and iii, white arrow). Smad9 expression was also observed in cranial nerves (orange arrow), mid brain (MB) and subpopulations of epidermal cells (EC). (**B**) Ventral view of the lower jaw (5 dpf) showing continued expression of Smad9 at the joint symphyses (orange arrows) and more dorsally located ethmoid plate (blue arrow). Inset (i) shows the MC and PQ symphysis with Smad9 expression not overlapping type II collagen expression (orange arrow). (**C**) Expression of *runx2a* was predominantly, but not exclusively, seen at the top (orange arrow) and posterior edge (blue arrow) in the operculum (green dotted line) by using in situ hybridisation. (**D**) Visualisation of proliferation by EdU click-iT predominantly showed proliferation adjacent to Smad9 expression near the top (orange arrows) and posterior edge (blue arrows) of the operculum. BR, branchiostegal ray; CH, ceratohyal; CL, cleithrum; E, eye; EC, epidermal cell; EP, Ethmoid plate; HS, hyosymplectic cartilage; MB, mid brain; MC, Meckel’s cartilage; OC, otic cartilage; OP operculum; OT, otolith; PQ, palatoquadrate. Scale bar is 50 µm.

The Smad9 protein sequence is highly conserved over vertebrate evolution (83.5% between human and zebrafish) and showed striking sequence similarity in the MH1 domain, MH2 domain, and the C-terminal SSVS BMPR phosphorylation motif, whereas the linker domain (between MH1 and MH2 domains) exhibited more sequence variability between human, mouse and zebrafish (Figure S2). This gave us further confidence that zebrafish Smad9 protein function is likely to be highly similar to land vertebrates. We have previously reported that Smad9 protein is expressed in the developing zebrafish skeleton at 6 and 7 dpf [9], but its expression profile prior to this is unknown. Therefore, initially we determined where Smad9 is expressed during key stages in the development of the zebrafish larval skeleton. Early craniofacial development of the lower jaw depends on neural crest cell migration during embryonic development, and these neural crest cells highly express *sox10* as a marker. At 30 h post-fertilisation (hpf), Smad9 was highly expressed around the pharyngeal pouches which give rise to lower jaw craniofacial elements, in a pattern that is complementary to, but largely not overlapping with *sox10* expression (Figure 1A). Note that *sox10*^+^ cells also weakly expressed Smad9 and we observed expression in the midbrain at 30 hpf (Figure 1A) that reduced over time (compare Figure 1A to Figure 2A). By 48 hpf, Smad9 was seen to be strongly expressed in the olfactory placode and in the musculoskeletal system where expression is observed at the anterior border of the migrating second arch which go on to form the ceratohyal (CH) [16] and at the developing symphysis of the Meckel’s cartilage (MC) (Figure 1B). Moreover, adjacent expression of Smad9 was also observed to enclose the more posterior pharyngeal pouches that give rise to ceratobranchial arches. Weak expression is also seen in the ocular muscles. Therefore, Smad9 is predominantly expressed in a subset of skeletal elements.

### 3.2. Smad9 Is Expressed within Joints and at Cartilage-Bone Interfaces during Early Jaw Development

Next, we examined Smad9 expression during at 4 dpf as the lower jaw skeletal elements including joint sites have fully formed. While Smad9 shows some expression in non-skeletal tissues, such as in the skin epithelium and cranial nerves, expression is concentrated in the developing skeletal system (Figure 2A). Smad9 is strongly expressed in pockets of cells located at the interface between cartilage and bone. These pockets of cells are situated between the palatoquadrate (PQ) and branchiostegal ray, and between the hyosymplectic and the operculum (Figure 2A). In the lower jaw at 5 dpf, Smad9 is co-expressed adjacent to type II Collagen producing chondrocytes at all cartilage joint sites such as the jaw joint between the MC and palatoquadrate (PQ), and in the midline at the MC symphysis and CH joint (Figure 2B).

As many of these skeletal sites are where skeletal precursors are located and where new cells are added to the skeletal elements, we looked at whether these cells co-expressed other early osteochondral markers and whether they were proliferative. In the jaw joint and at the midline joints of the MC symphysis and CH, Smad9 was strongly expressed in cells which were positive for *sox10* but negative for type II Collagen (Figure S3), with only weak expression of Smad9 in type II Collagen expressing cells, suggesting that Smad9 is downregulated in cells as they differentiate and become mature chondrocytes. We next tested whether the Smad9 expression overlapped with expression of the early osteochondral gene transcription factor *runx2a*, which plays a key role in differentiation to both the chondrocyte and osteoblast fates [40]. While *runx2a* is expressed throughout the operculum, in positions corresponding with the surface osteoblasts there is little colocalisation with the pocket of cells at the neck (characteristic pan handle shape) of the operculum (Figure 2C), suggesting that Smad9 is expressed prior to *runx2a* in skeletal precursor cells. To test whether Smad9^+^ cells are proliferative, we labelled fish with the thymidine analogue EdU which is incorporated during DNA replication in proliferation. We observed that a proportion of proliferating cells overlapped those with Smad9 protein (Figure 2D). From these data we conclude that Smad9 protein is dynamically produced in an osteochondral precursor population and that protein levels are reduced as cells differentiate to an osteoblast or chondrocyte fate.

As Smad9 is an inhibitory transcription factor of the BMP family, we next sought to establish the relationship between Smad9 and BMP signalling in skeletal precursors. We therefore tested whether Smad9 was seen in cells responding to BMP signalling, by making use of a transgenic line carrying BMP responsive elements (BMPre) driving GFP expression (Figure S4A,B), and by labelling larvae with antibodies raised against Smad1 (Figure S4C,D) and Smad5 (Figure S4E–G) at 7 dpf. We observed little overlap between Smad9 protein and expression of the *BMPre:GFP* reporter, as we mostly observed GFP expression in the cranial musculature whilst Smad9 was absent (Figure S4A,B); therefore fitting with the likely role of Smad9 being a repressor of BMP signalling. By contrast, Smad1 showed a similar pattern of expression to the *BMPre:GFP* reporter, with both most strongly expressed in the craniofacial skeletal musculature (Figure S4C,D). Smad5 exhibited weaker expression in the muscle than Smad1, but strongest expression appeared to be in the endothelial cells of the vascular system, with little or no expression in the skeletal precursors (Figure S4E–G). We observed similar expression patterns in the trunk, where Smad1 was expressed in the musculature (Figure S5A), Smad5 in the musculature and vascular endothelial cells (Figure S5B), and Smad9 in notochord sheath cells (Figure S5C). From this we concluded that Smad9 protein largely did not colocalise with Smad1, Smad5 or cells responding to BMP signalling, consistent with its function as a repressor of BMP signalling.

### 3.3. Pharmacological Manipulation Modulates Osteochondral Smad9 Expression at Skeletal Sites

As our data suggests that Smad9 labels a population of osteochondral precursors, we next wanted to test how this population of cells would respond to pharmacological treatment with drugs known to act on the skeleton. We selected three well characterised compounds. Prednisolone (PD) is widely reported to induce glucocorticoid induced osteoporosis (GIOP) in many species and to diminish osteoblast activation following loading [41]. In zebrafish specifically, larval treatment with PD leads to reduced mineralisation [25], while in adults, treatment leads to impaired skeletal regeneration [42,43]. Retinoid acid (RA) plays a complex role in the skeleton, as a key factor in morphogenesis it is controlling precursor cell fate by forming a tightly controlled gradient. For example, mutations in genes which control the inactivation of RA such as *cyp261* have been demonstrated to lead to ectopic and early entry to the osteocyte fate leading to premature ossification of the spinal column and of suture osteoblasts causing craniosynostosis [44,45]. We also selected dorsomorphin (DM) as an inhibitor of the BMP pathway, which was discovered in a zebrafish screen and has been shown to inhibit phosphorylation of R-Smads [23]. As we observed strong expression of Smad9 in locations associated with skeletal precursors, we decided to treat larvae for 48-h with the chosen biomolecules with dosages confirmed after titration (Figure S1) between 3 and 5 dpf, as by 3 dpf embryonic skeletal patterning has concluded but skeletal growth and morphogenesis are ongoing.

**Figure 3:**
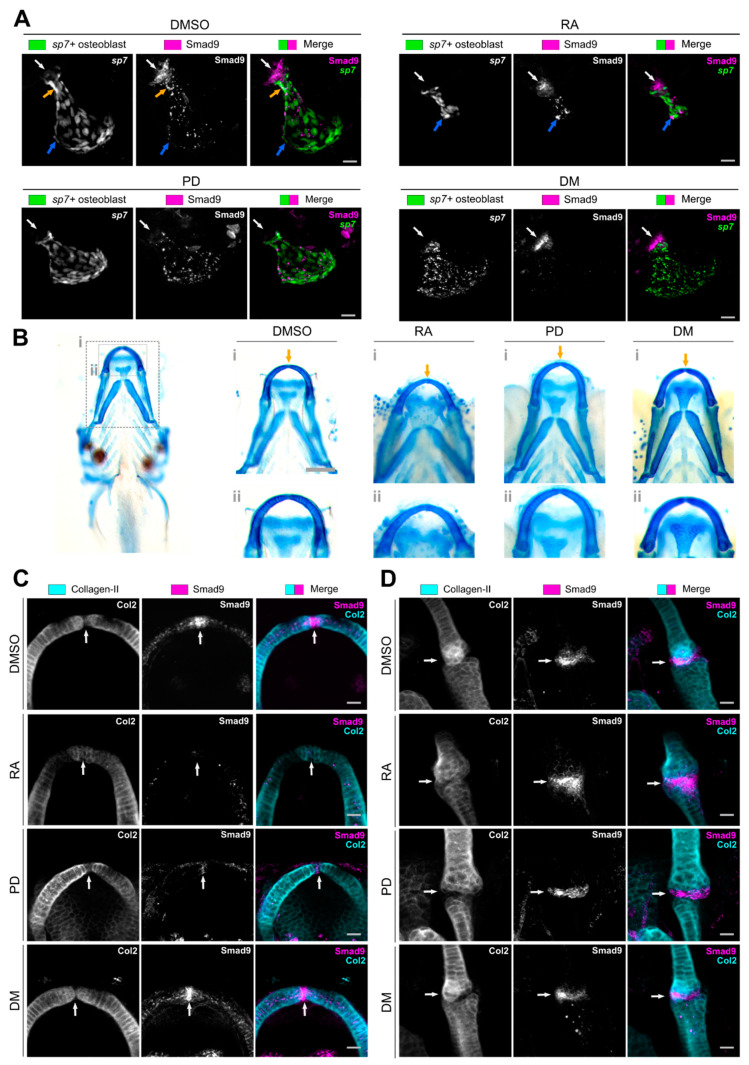
Pharmacological treatment alters Smad9 expression in line with changes to skeletal morphology: (**A**) Lateral view of the operculum at 5.25 dpf following 48 h treatment with either: DMSO (control), 1 µM retinoic acid (RA), 25 µM prednisolone (PD) or 4 µM dorsomorphin (DM). Control larvae show Smad9 expression in the pocket of cells at the top of the operculum (white arrow) and punctuate expression in the neck (orange arrow) and ventral edge of the operculum (blue arrow). PD treatment dramatically reduced Smad9 expression in the pocket of cells at the top of the operculum (white arrow) and increased punctate expression. RA treatment reduced osteoblast number and operculum outgrowth; bright Smad9 expression was seen at the ventral edge of the operculum (blue arrow). DM treatment had little effect on Smad9 expression at the top of the operculum (though there was a reduction in punctae in the lower operculum) or on osteoblast number. Scale bar is 20 µm. (**B**) Alcian blue staining of larvae treated with DMSO, RA, PD or DM at 5 dpf showing the ventral jaw (inset i) and MC (inset ii). RA treatment resulted in deformed cartilage growth at the midline in MC, while PD and DM did not lead to dramatic changes to morphology (orange arrow). Scale bar is 100 µm. (**C**) Ventral views of MC at 5.25 dpf labelled for type II Collagen and Smad9 in control and drug treated larvae. Controls showed bright Smad9 expression at the midline in the MC with a gradient of expression throughout the cartilage. RA treatment resulted in almost complete loss of Smad9 expression at the midline (white arrows). PD led to reduced expression of the Smad9 (white arrow), while DM treatment had little effect on Smad9 expression at the midline. (**D**) Ventral views of the MC-PQ synovial joint at 5.25 dpf labelled for type II Collagen and Smad9 in control and drug treated larvae. Smad9 expression is maintained in the joint space in all conditions (white arrow). Scale bar is 20 µm.

Treatment of larvae from 3–5 dpf with RA led to a dramatic decrease in osteoblast number, and a reduction in the mineralised area of the operculum similar to that described for the *cyp26b1* mutant (Figure 3A) [46]. Treatment with RA led to altered expression of Smad9, such that the pocket of cells at the cartilage bone interface were still labelled, but that there were fewer cells in this location, and within osteoblasts expression was patchy with some bright Smad9^+^ cells at the ventral edge of the operculum (Figure 3A). In the lower jaw cartilages, RA treatment led to altered cartilage growth at the midline in the MC symphysis similar to that previously described in the *cyp26b1* mutant [46], with subtle changes to cartilage morphology at the joint, close to where the retroarticular process (RAP) will form, reminiscent of changes observed at the joint following immobilisation (Figure 3B,C) [47]. Correspondingly, we saw an almost complete loss of Smad9 expression at the MC symphysis (Figure 3C), where both the bright cells and the more punctate gradient in chondrocytes were both lost. At the joint we observed more subtle changes to distribution of Smad9 with expression observed in the PQ as well as the joint interzone (Figure 3D).

Treatment of larvae with PD led to a substantial reduction of Smad9 in the pocket between the hyosymplectic and the operculum, such that it was almost completely absent with only punctate vesicular expression in the osteoblasts covering the operculum remaining (Figure 3A). In the cartilage overall morphology of the elements was unchanged (Figure 3B), although cells at the midline in the MC symphysis appeared somewhat larger; expression of Smad9 at the midline in the MC symphysis was reduced, such that there were no bright cells at the midline and only punctate expression in the cartilage (Figure 3C). At the jaw joint, both cartilage morphology and Smad9 expression appeared largely unchanged (Figure 3D).

By contrast, treatment with DM led to no dramatic differences to Smad9 expression and skeletogenesis. In the operculum we observed only subtle changes to morphology, and a slight change to the distribution of the Smad9^+^ cells pocket and its relationship with the surrounding osteoblasts (Figure 3A). In the cartilage we observed no change to overall morphology, and a slight enhancement of Smad9 expression at the joint sites (Figure 3B–D). During skeletal cell maturation, the Wnt pathway plays an important role in joint formation and its expression is dependent on joint loading [47]. The BMP pathway has been shown to negatively regulate Wnt at skeletal sites [48]. To test whether DM treatment had an effect Wnt expression near joint sites we used a canonical Wnt transgenic reporter line to visualise reporter expression at the lower jaw joint sites. Firstly, we did not observe any co-expression of the *Wnt:GFP* reporter and Smad9 at the joint symphyses. DM treatment led to a decrease in reporter activity both at the midline MC symphysis (Figure S6A) and at the MC-PQ symphysis (Figure S6B) whilst Smad9 expression appeared not affected.

We therefore provide evidence that DM has a limited effect on the Smad9^+^ population of cells, that PD treatment leads to a reduction in bright Smad9^+^ expressing cells in both the cartilage and the bone. RA treatment led to stronger phenotypes as we also observed a loss of Smad9 expression at the MC symphysis midline causing ectopic cartilage formation. In the operculum, the reduced Smad9 expression correlated with a reduction in osteoblast number.

### 3.4. Osteoblast Ablation Induces Increased Expression of Smad9 during Larval De Novo Osteoblast Formation

**Figure 4:**
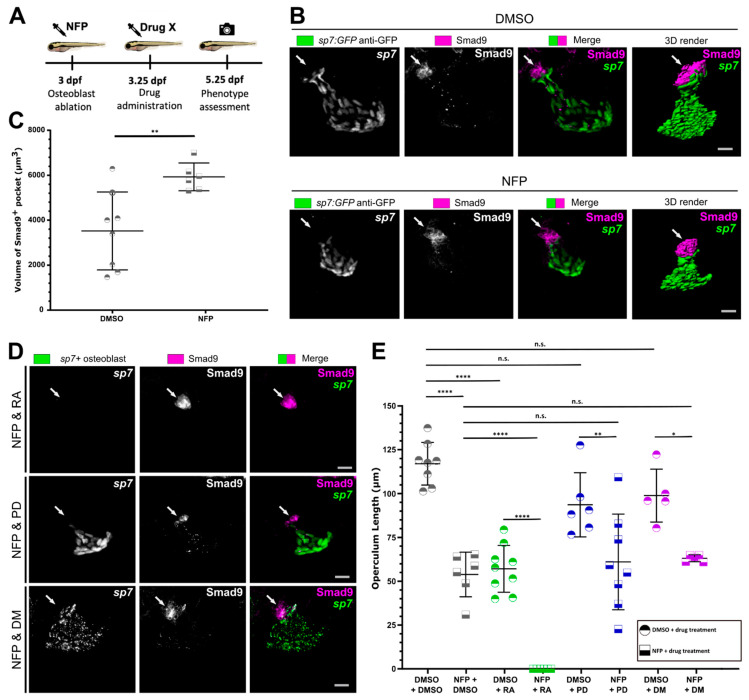
Osteoblast ablation and pharmacological treatment affects osteoblast generation and Smad9 expression: (**A**) Schematic showing that osteoblasts were ablated with nifurpirnol (NFP) at 3 dpf; followed by drug treatment (for 48 h) and phenotype assessment at 5.25 dpf. (**B**) Lateral view of the operculum after 6 h DMSO or NFP treatment. NFP treatment resulted in a smaller regenerated operculum and expanded Smad9 expression in the pocket of cells at the top of the operculum (white arrow). (**C**) Analysis of 3D rendered images showed NFP treatment significantly increased the volume of the Smad9^+^ pocket of cells. (**D**) Lateral view of operculum after a combination of NFP and RA, PD or DM treatment. Treatment with NFP and RA blocked osteoblast regeneration but maintained Smad9 expression. NFP and PD treatment results in a depleted Smad9 expression but a regenerated operculum. NFP and DM treatment had no change to Smad9 expression (white arrow) or operculum regeneration. (**E**) Quantification of operculum length after drug treatments at 5.25 dpf. N ≥ 5. Scale bar is 20 µm. ^ns^ *p* > 0.05, * *p* ≤ 0.05, ** *p* ≤ 0.01, **** *p* ≤ 0.0001.

We then wanted to test whether treatment with the biomolecules would prevent differentiation of new osteoblasts following ablation of existing osteoblasts at 3 dpf. We therefore first ablated osteoblasts with nifurpirinol (NFP), which was then followed by RA, PD or DM treatment for 48-h after ablation (Figure 4A). Bergemann et al. [49] demonstrated that a less toxic antibiotic NFP can be used to effectively ablate osteoblasts in the *sp7:mCherry-NTR* transgenic line. We confirmed that treating *sp7:mCherry-NTR* transgenic larvae with NFP successfully ablated osteoblasts at the times of interest. Larvae were treated for 6 h at 3 dpf with NFP, and by 24 h post treatment (hpt) full ablation of the osteoblasts in the operculum was seen (Figure S7A,B). Macrophages were observed to engulf the remnants of *sp7*+ osteoblasts (Figure S7C and Movie S1). We therefore used this model of treatment with NFP for 6 h to ablate osteoblasts on day 3 (from 3 dpf to 3.25 dpf) of development. A rapid recovery of osteoblasts in the operculum was seen, as already 48 h after *sp7*+ osteoblasts were clearly apparent organised in the characteristic fan shape of the operculum. The regenerated operculum was significantly smaller than the control at the same point in development (48 hpt/5 dpf) (Figure S7B). The size of the regenerated operculum correlates to the size of the operculum in a younger untreated larva, suggesting that the operculum regenerates following the ontogenetic pattern. We then determined whether the Smad9^+^ pocket was present after ablation and whether these cells would contribute to regeneration following the ablation of *sp7* expressing osteoblasts. Strikingly, we observed an expansion of the Smad9^+^ pocket indicating that this pool is activated when osteoblast repopulation is occurring (Figure 4B,C).

By deploying this model, we were able to test the hypothesis that if RA was preventing new osteoblast formation, we would see impaired osteoblast repopulation after ablation. Indeed, we observed that while Smad9 expression was still clearly observed in the pocket of cells, no *sp7*+ osteoblasts were seen following treatment (Figure 4D) and regrowth of the operculum was completely absent (Figure 4E). Treatment with PD and DM following NFP ablation of osteoblasts lead to no obvious additional defects to operculum size over ablation alone (Figure 4D,E), indicating that early acute osteoblast regenerative capacity in larvae is not dependent on glucocorticoid and BMP signalling. However, we observed that after osteoblast ablation, both the Smad9^+^ pocket and *sp7*+ osteoblast operculum size (*p* = 0.12) showed a smaller tendency in PD treated larvae (Figure 4D,E), implying that PD treatment had a limited effect on the Smad9^+^ cell population.

Taken together these data show that Smad9 is expressed in precursor populations in both cartilage and bone elements, concentrated at interfaces. We show that pharmacological manipulations known to affect osteochondral cells were able to modulate Smad9 expression at these sites. After osteoblast depletion we observed an increased Smad9^+^ pocket indicating this pool provides cells for osteoblast repopulation. We identified striking changes to Smad9 expression upon RA treatment correlating with altered midline chondrocyte differentiation and overgrowth at the MC symphysis in cartilage and with rapid osteoblast differentiation limiting operculum growth and completely preventing regrowth following ablation. PD treatment led to a notable reduction of Smad9 MC symphysis expression and in the Smad9^+^ pocket at the dorsal tip of the operculum. In line with DM being a BMP signalling inhibitor, DM had a limited effect on Smad9 expression and osteochondral populations, which further validates Smad9 acting as a transcriptional repressor of BMP signalling in zebrafish as well.

## 4. Discussion

Here we describe the expression of Smad9 (previously known as Smad8) during early skeletal development. We show that during skeletal development expression of Smad9 largely does not colocalise with Smad1, Smad5 or with cells responding to BMP signalling. We show that Smad9 labels an osteochondral precursor population, which is proliferative and expands during regeneration following ablation of *sp7*+ osteoblasts. Murine Smad9 has been shown to be expressed in distal and proximal sites of skeletal elements in developing skeletal elements [50]. Our data shows a similar expression pattern during early development and that this expression becomes limited to a subset of precursor cells manipulation of which could lead to alterations in skeletal patterning. It has previously been shown that Smad9 can be induced by BMP7, as when beads coated with BMP7 were implanted into chick hind limbs, the expression domain of Smad9 was increased [51]. Here we expand on this data by showing how Smad9 is differentially regulated by RA and PD, but is largely unchanged following treatment with the BMP signalling inhibitor DM.

While over the last decade there has been a huge increase in the number of GWAS associated loci harbouring potential novel genes with skeletal phenotypes, such as eBMD [52], OP [53] and fracture susceptibility [54], there remains an unmet need for rapid downstream functional characterisation [55]. Studies of rare bone diseases associated with HBM and subsequent detailed functional characterisation of identified associated genes, such as the Wnt regulator SOST, have underpinned the development of the new OP therapeutic Romozsumab, an antibody that blocks *SOST* function leading to bone anabolism [56,57]. Similar to SOST, a damaging mutation predicted to affect SMAD9 function has been identified in a pedigree with HBM [9]. As Smad9 is a R-SMAD and a downstream transcriptional inhibitor of the SMAD-dependent BMP signalling pathway, it represents an appealing target for pharmacological manipulation. Dysregulation of BMP signalling is associated with many disease pathologies, an example of which is the ectopic bone forming disease fibrodysplasia ossificans progressivia (FOP) where a gain of function mutation in type II BMPR (ACVR1) leads to constitutively active kinase domain enhancing R-SMAD translocation to the nucleus [58]. A number of therapeutics based on modulation of BMP signalling are now licensed for clinical use in skeletal disease and orthopedic settings [59]. Zebrafish are increasingly used in functional characterisation of genes identified by GWAS and in drug discovery pipelines. Indeed, the BMP inhibitor DM was first identified by its dorsalising effects in embryonic zebrafish [12], and analogues of DM that can target Acvr1 are now repurposed (from being a leukaemia therapy) and in clinical trials to prevent the ectopic mineralisation seen in FOP [24]. Drug screens in zebrafish can therefore be a leading platform to discover new biologically active agents. However, subtle physiological differences between mammalian and teleost bone should be taken into account when interpreting results for certain drug targets. For example, unlike human bone, zebrafish lack hematopoietic bone marrow and show trabeculation only in some bones. These differences need to be considered when especially investigating potential drug targets that focus on trabeculation and bone marrow integrity rather than osteoanabolic cell response. When focussing on amending osteoblast function to discover putative osteoactive biomolecules that could act on validated HBM genes, assays could be performed in larvae [60], on ex vivo cultured elasmoid scales [28,29], or administered to adult fish to test effects on the skeleton during regeneration/bone healing in exoskeletal elements [43].

Firstly, for any newly identified HBM gene, it is important to validate that it is expressed in the developing skeleton and shows functional relevance. Here we show that Smad9 protein is indeed dynamically expressed during early skeletal development. During development progression, Smad9 expression is becoming more restricted to precursor populations in the cartilage, at joint sites where new cells are added, and in pockets of cells at the interface between cartilage and bone. This population of cells expands during regeneration of the operculum following osteoblast ablation. Previous mouse studies using a transgenic *LacZ* reporter lines under control of an endogenous Smad9 promoter detected *LacZ* expression in the developing skeleton (e.g., ribs, maxilla, mandible), gut, hind brain, and trachea [50]. This study shows for the first time that Smad9 is expressed at symphyses of cartilage and bone. Moreover, we show that there is little overlap in expression between Smad9 and the other BMP dependent R-Smads (Smad1 and Smad5) or with cells responding to BMP signalling, consistent with a role for Smad9 functioning to inhibit BMP signalling. We saw little difference to Smad9 expression or to skeletal patterning following treatment with DM, consistent with Smad9 already acting to repress BMP signalling at these sites. It is therefore interesting to note that these findings are in line with the previous evidence that the damaging p.Pro22Leu mutation in *SMAD9* does not cause FOP in humans, as in developing zebrafish it is not expressed in FOP prone and BMP signalling responsive tissues such as muscle.

Treatment of larvae with PD led to an almost complete loss of Smad9 in the precursor pocket of the operculum, and diminished Smad9 expression at cartilage joint sites. Glucocorticoids have been associated with enhanced chondrogenic differentiation from mesenchymal stem cells [61]. However long-term use of PD is associated with OP, and osteonecrosis [62] and has been also shown to diminish skeletal regeneration following injury in zebrafish due to dampening of the inflammation response that is important for bone remodelling and tissue repair [27]. Our data showed that following PD treatment the Smad9^+^ pools were diminshed but that after osteoblast ablation a small pool of Smad9+ cells remained. This is likely due to the fact that during ablation the Smad9^+^ pool expands which could explain why PD treatment had a little effect on operculum regrowth, as these remaining Smad9^+^ cells may repopulate the operculum with new osteoblasts. Another factor may be that the inflammation response is not crucial for repleting the osteoblast pool. While we saw relatively minor effects on bone following PD treatment, this is likely because at least in part osteoporotic phenotypes associated with PD treatment are associated with increased osteoclast action and altered metabolism, at the stages studied here zebrafish have not yet formed active osteoclasts (they become active around 10 dpf [21]) and the metabolic changes that occur on changing from yolk feeding to free feeding have also not yet taken place.

Treatment of larvae with RA led to a loss of Smad9 at the midline; excess RA is associated with cartilage overgrowth leading to craniosynostosis and jaw mispatterning in *cyp26b1* mutants in which the degradation of RA is altered [44], again consistent with Smad9 normally acting to prevent premature chondrogenesis. In the spine treatment with RA during spine patterning is associated with ectopic bone formation [36]. Interestingly, we observed Smad9 expression in the notochord sheath, though we did not study expression of notochord Smad9 after RA treatment. However, in the operculum, treatment with RA led to reduced osteoblast differentiation and a smaller operculum size. Following ablation treatment with RA led to a complete failure of osteoblast differentiation, such that the Smad9^+^ cells were present, but no new osteoblasts were formed. Since Smad9 expression was altered in response to pharmacological treatment and osteoblast ablation, future research should focus on loss-of function *smad9* alleles ideally combined with transcriptomic or proteomic analysis to determine the downstream effects of changed Smad9 expression.

Taken together the fact that Smad9 expression is predictive of the differentiation of osteochondral precursor populations suggests that it plays a role controlling the timing of differentiation. Smad9 could therefore both be a useful read out of regenerative capacity in the skeleton and an appropriate target for future pharmacological manipulation relevant to developing osteoanabolic therapies.

## Data Availability

Raw data will be made available on the data.bris repository with permanent DOI fixed upon manuscript acceptance.

## Supplementary Materials

The following are available online at https://www.mdpi.com/2218-273X/11/2/277/s1, Figure S1: Dose response of pharmacological treatments; Figure S2: Smad9 protein is highly conserved between zebrafish and land mammals; Figure S3: Smad9 is co-expressed with Sox10 at joint sites; Figure S4: Smad9 expression does not overlap expression of BMP responsive cells, Smad1 or Smad5; Figure S5: Smad9 is expressed in the notochord whilst Smad1, Smad5 and the BMPre:GFP transgene are not; Figure S6: Pharmacological manipulation effects Wnt signalling in larvae jaws; Figure S7: Chemical ablation of osteoblasts induces rapid macrophage clearance of apoptotic cells and subsequent regeneration of the operculum resident osteoblasts; Movie S1: Rapid clearance of sp7+ cells by macrophages after chemical ablation.

## References

[1] Feng X., McDonald J.M. (2011). Disorders of bone remodeling. Annu. Rev. Pathol. Mech. Dis..

[2] Hernlund E., Svedbom A., Ivergård M., Compston J., Cooper C., Stenmark J., McCloskey E.V., Jonsson B., Kanis J.A. (2013). Osteoporosis in the European Union: Medical management, epidemiology and economic burden. Arch. Osteoporos..

[3] Svedbom A., Hernlund E., Ivergård M., Compston J., Cooper C., Stenmark J., McCloskey E.V., Jönsson B., Kanis J.A. (2013). Osteoporosis in the European Union: A compendium of country-specific reports. Arch. Osteoporos..

[4] Metcalf L.M., Aspray T.J., McCloskey E.V. (2017). The effects of parathyroid hormone peptides on the peripheral skeleton of postmenopausal women. A systematic review. Bone.

[5] Watanabe A., Yoneyama S., Nakajima M., Sato N., Takao-Kawabata R., Isogai Y., Sakurai-Tanikawa A., Higuchi K., Shimoi A., Yamatoya H. (2012). Osteosarcoma in Sprague-Dawley rats after long-term treatment with teriparatide (human parathyroid hormone (1–34)). J. Toxicol. Sci..

[6] Gregson C.L., Steel S.A., O’Rourke K.P., Allan K., Ayuk J., Bhalla A., Clunie G., Crabtree N., Fogelman I., Goodby A. (2012). “Sink or swim”: An evaluation of the clinical characteristics of individuals with high bone mass. Osteoporos. Int..

[7] McClung M.R., Grauer A., Boonen S., Bolognese M.A., Brown J.P., Diez-Perez A., Langdahl B.L., Reginster J.-Y., Zanchetta J.R., Wasserman S.M. (2014). Romosozumab in postmenopausal women with low bone mineral density. N. Engl. J. Med..

[8] Balemans W. (2001). Increased bone density in sclerosteosis is due to the deficiency of a novel secreted protein (SOST). Hum. Mol. Genet..

[9] Gregson C.L., Bergen D.J.M., Leo P., Sessions R.B., Wheeler L., Hartley A., Youlten S., Croucher P., McInerney-Leo A.M., Fraser W. (2020). A Rare Mutation in SMAD9 Associated with High Bone Mass Identifies the SMAD-Dependent BMP Signaling Pathway as a Potential Anabolic Target for Osteoporosis. J. Bone Miner. Res..

[10] Nakao A., Imamura T., Souchelnytskyi S., Kawabata M., Ishisaki A., Oeda E., Tamaki K., Hanai J.-I., Heldin C.-C., Miyazono K. (1997). TGF-β receptor-mediated signalling through Smad2, Smad3 and Smad4. EMBO J..

[11] Tsukamoto S., Mizuta T., Fujimoto M., Ohte S., Osawa K., Miyamoto A., Yoneyama K., Murata E., Machiya A., Jimi E. (2014). Smad9 is a new type of transcriptional regulator in bone morphogenetic protein signaling. Sci. Rep..

[12] Yu P.B., Hong C.C., Sachidanandan C., Babitt J.L., Deng D.Y., Hoyng S.A., Lin H.Y., Bloch K.D., Peterson R.T. (2008). Dorsomorphin inhibits BMP signals required for embryogenesis and iron metabolism. Nat. Chem. Biol..

[13] Howe K., Clark M.D., Torroja C.F., Torrance J., Berthelot C., Muffato M., Collins J.E., Humphray S., McLaren K., Matthews L. (2013). The zebrafish reference genome sequence and its relationship to the human genome. Nature.

[14] Askary A., Smeeton J., Paul S., Schindler S., Braasch I., Ellis N.A., Postlethwait J., Miller C.T., Crump J.G. (2016). Ancient origin of lubricated joints in bony vertebrates. eLife.

[15] Kimmel C.B., Miller C.T., Moens C.B. (2001). Specification and morphogenesis of the zebrafish larval head skeleton. Dev. Biol..

[16] Eames B.F., DeLaurier A., Ullmann B., Huycke T.R., Nichols J.T., Dowd J., McFadden M., Sasaki M.M., Kimmel C.B. (2013). FishFace: Interactive atlas of zebrafish craniofacial development at cellular resolution. BMC Dev. Biol..

[17] MacRae C.A., Peterson R.T. (2015). Zebrafish as tools for drug discovery. Nature Reviews Drug Discovery.

[18] Lleras-Forero L., Winkler C., Schulte-Merker S. (2020). Zebrafish and Medaka as Models for Biomedical Research of Bone Diseases.

[19] Singh S.P., Holdway J.E., Poss K.D. (2012). Regeneration of Amputated Zebrafish Fin Rays from De Novo Osteoblasts. Dev. Cell.

[20] Mantoku A., Chatani M., Aono K., Inohaya K., Kudo A. (2016). Osteoblast and osteoclast behaviors in the turnover of attachment bones during medaka tooth replacement. Dev. Biol..

[21] Witten P.E., Huysseune A. (2009). A comparative view on mechanisms and functions of skeletal remodelling in teleost fish, with special emphasis on osteoclasts and their function. Biol. Rev..

[22] Hammond C.L., Schulte-Merker S. (2009). Two populations of endochondral osteoblasts with differential sensitivity to Hedgehog signalling. Development.

[23] Chen X., Wang Z., Duan N., Zhu G., Schwarz E.M., Xie C. (2018). Osteoblast–osteoclast interactions. Connect. Tissue Res..

[24] Yu P.B., Deng D.Y., Lai C.S., Hong C.C., Cuny G.D., Bouxsein M.L., Hond D.W., McManus P.M., Katagiri T., Sachidanandan C. (2008). BMP type I receptor inhibition reduces heterotopic ossification. Nat. Med..

[25] He H., Wang C., Tang Q., Yang F., Xu Y. (2018). Possible mechanisms of prednisolone-induced osteoporosis in zebrafish larva. Biomed. Pharmacother..

[26] Pasqualetti S., Congiu T., Banfi G., Mariotti M. (2015). Alendronate rescued osteoporotic phenotype in a model of glucocorticoid-induced osteoporosis in adult zebrafish scale. Int. J. Exp. Pathol..

[27] Geurtzen K., Vernet A., Freidin A., Rauner M., Hofbauer L.C., Schneider J.E., Brand M., Knopf F. (2017). Immune Suppressive and Bone Inhibitory Effects of Prednisolone in Growing and Regenerating Zebrafish Tissues. J. Bone Miner. Res..

[28] Bergen D.J.M., Kague E., Hammond C.L. (2019). Zebrafish as an emerging model for osteoporosis: A primary testing platform for screening new osteo-active compounds. Front. Endocrinol..

[29] De Vrieze E., Zethof J., Schulte-Merker S., Flik G., Metz J.R. (2015). Identification of novel osteogenic compounds by an ex-vivo sp7:luciferase zebrafish scale assay. Bone.

[30] Aleström P., D’Angelo L., Midtlyng P.J., Schorderet D.F., Schulte-Merker S., Sohm F., Warner S. (2020). Zebrafish: Housing and husbandry recommendations. Lab. Anim..

[31] DeLaurier A., Frank Eames B., Blanco-Sánchez B., Peng G., He X., Swartz M.E., Ullmann B., Westerfield M., Kimmel C.B. (2010). Zebrafish sp7:EGFP: A transgenic for studying otic vesicle formation, skeletogenesis, and bone regeneration. Genesis.

[32] Alexander C., Zuniga E., Blitz I.L., Wada N., le Pabic P., Javidan Y., Zhang T., Cho K.W., Crump J.G., Schilling T.F. (2011). Combinatorial roles for BMPs and endothelin 1 in patterning the dorsal-ventral axis of the craniofacial skeleton. Development.

[33] Dutton J.R., Antonellis A., Carney T.J., Rodrigues F.S.L.M., Pavan W.J., Ward A., Kelsh R.N. (2008). An evolutionarily conserved intronic region controls the spatiotemporal expression of the transcription factor Sox10. BMC Dev. Biol..

[34] Moro E., Ozhan-Kizil G., Mongera A., Beis D., Wierzbicki C., Young R.M., Bournele D., Domenichini A., Valdivia L.E., Lum L. (2012). In vivo Wnt signaling tracing through a transgenic biosensor fish reveals novel activity domains. Dev. Biol..

[35] Dee C.T., Nagaraju R.T., Athanasiadis E.I., Gray C., del Ama L.F., Johnston S.A., Secombes C.J., Cvejic A., Hurlstone A.F.L. (2016). CD4-Transgenic Zebrafish Reveal Tissue-Resident Th2- and Regulatory T Cell–like Populations and Diverse Mononuclear Phagocytes. J. Immunol..

[36] Spoorendonk K.M., Peterson-Maduro J., Renn J., Trowe T., Kranenbarg S., Winkler C., Schulte-Merker S. (2008). Retinoic acid and Cyp26b1 are critical regulators of osteogenesis in the axial skeleton. Development.

[37] Huo L., Wang L., Yang Z., Li P., Geng D., Xu Y. (2018). Prednisolone induces osteoporosis-like phenotypes via focal adhesion signaling pathway in zebrafish larvae. Biol. Open..

[38] Schindelin J., Arganda-Carreras I., Frise E., Kaynig V., Longair M., Pietzsch T., Preibisch S., Rueden C., Saalfeld S., Schmid B. (2012). Fiji: An open-source platform for biological-image analysis. Nat. Methods.

[39] Lassmann T., Frings O., Sonnhammer E.L.L. (2009). Kalign2: High-performance multiple alignment of protein and nucleotide sequences allowing external features. Nucleic Acids Res..

[40] Fujita T., Azuma Y., Fukuyama R., Hattori Y., Yoshida C., Koida M., Ogita K., Komori T. (2004). Runx2 induces osteoblast and chondrocyte differentiation and enhances their migration by coupling with PI3K-Akt signaling. J. Cell Biol..

[41] Bergström I., Isaksson H., Koskela A., Tuukkanen J., Ohlsson C., Andersson G., Windahl S. (2018). Prednisolone treatment reduces the osteogenic effects of loading in mice. Bone.

[42] De Vrieze E., Van Kessel M.A.H.J., Peters H.M., Spanings F.A.T., Flik G., Metz J.R. (2014). Prednisolone induces osteoporosis-like phenotype in regenerating zebrafish scales. Osteoporos. Int..

[43] Schmidt J.R., Geurtzen K., von Bergen M., Schubert K., Knopf F. (2019). Glucocorticoid Treatment Leads to Aberrant Ion and Macromolecular Transport in Regenerating Zebrafish Fins. Front. Endocrinol..

[44] Laue K., Pogoda H.M., Daniel P.B., Van Haeringen A., Alanay Y., Von Ameln S., Rachwalski M., Morgan T., Gray M.J., Breuning M.H. (2011). Craniosynostosis and multiple skeletal anomalies in humans and zebrafish result from a defect in the localized degradation of retinoic acid. Am. J. Hum. Genet..

[45] Jeradi S., Hammerschmidt M. (2016). Retinoic acid-induced premature osteoblast-to-preosteocyte transitioning has multiple effects on calvarial development. Development.

[46] Laue K., Jänicke M., Plaster N., Sonntag C., Hammerschmidt M. (2008). Restriction of retinoic acid activity by Cyp26b1 is required for proper timing and patterning of osteogenesis during zebrafish development. Development.

[47] Brunt L.H., Begg K., Kague E., Cross S., Hammond C.L. (2017). Wnt signalling controls the response to mechanical loading during zebrafish joint development. Development.

[48] Kamiya N., Ye L., Kobayashi T., Mochida Y., Yamauchi M., Kronenberg H.M., Feng J.Q., Mishina Y. (2008). BMP signaling negatively regulates bone mass through sclerostin by inhibiting the canonical Wnt pathway. Development.

[49] Bergemann D., Massoz L., Bourdouxhe J., Carril Pardo C.A., Voz M.L., Peers B., Manfroid I. (2018). Nifurpirinol: A more potent and reliable substrate compared to metronidazole for nitroreductase-mediated cell ablations. Wound Repair Regen..

[50] Huang Z., Wang D., Ihida-Stansbury K., Jones P.L., Martin J.F. (2009). Defective pulmonary vascular remodeling in Smad8 mutant mice. Hum. Mol. Genet..

[51] Abarca-Buis R.F., Bustamante M., Cuervo R., Aguilar-Fernández-de-Lara D., Chimal-Monroy J. (2011). Smad8 is expressed in the anterior necrotic zone: Evidence for a role of bone morphogenetic proteins/SMAD signaling in the activation of a molecular cascade that culminates in cell death. Dev. Growth Differ..

[52] Kemp J.P., Morris J.A., Medina-Gomez C., Forgetta V., Warrington N.M., Youlten S.E., Zheng J., Gregson C.L., Grundberg E., Trajanoska K. (2017). Identification of 153 new loci associated with heel bone mineral density and functional involvement of GPC6 in osteoporosis. Nat. Genet..

[53] Morris J.A., Kemp J.P., Youlten S.E., Laurent L., Logan J.G., Chai R.C., Vulpescu N.A., Forgetta V., Kleinman A., Mohanty S.T. (2019). An atlas of genetic influences on osteoporosis in humans and mice. Nat. Genet..

[54] Trajanoska K., Rivadeneira F. (2019). The genetic architecture of osteoporosis and fracture risk. Bone.

[55] Tobias J.H., Duncan E.L., Kague E., Hammond C.L., Gregson C.L., Bassett J.H.D., Williams G.R., Min J.L., Gaunt T.R., Karasik D. (2020). Opportunities and challenges in functional genomics research in osteoporosis: Report from a workshop held by the Causes Working Group of the Osteoporosis and Bone Research Academy of the Royal Osteoporosis Society on October 5th 2020. Front. Endocrinol..

[56] Brunkow M.E., Gardner J.C., Van Ness J., Paeper B.W., Kovacevich B.R., Proll S., Skonier J.E., Zhao L., Sabo P.J., Fu Y.-H. (2001). Bone dysplasia sclerosteosis results from loss of the SOST gene product, a novel cystine knot-containing protein. Am. J. Hum. Genet..

[57] Cosman F., Crittenden D.B., Adachi J.D., Binkley N., Czerwinski E., Ferrari S., Hofbauer L.C., Lau E., Lewiecki E.M., Miyauchi A. (2016). Romosozumab treatment in postmenopausal women with osteoporosis. N. Engl. J. Med..

[58] Carney T.J., Feitosa N.M., Sonntag C., Slanchev K., Kluger J., Kiyozumi D., Gebauer J.M., Talbot J.C., Kimmel C.B., Sekiguchi K. (2010). Genetic analysis of fin development in zebrafish identifies furin and hemicentin1 as potential novel fraser syndrome disease genes. PLoS Genet..

[59] Lowery J.W., Rosen V. (2018). Bone morphogenetic protein–based therapeutic approaches. Cold Spring Harb. Perspect. Biol..

[60] Chen J.R., Lai Y.H., Tsai J.J., Hsiao C.D. (2017). Live fluorescent staining platform for drug-screening and mechanism-analysis in zebrafish for bone mineralization. Molecules.

[61] Shintani N., Hunziker E.B. (2011). Differential effects of dexamethasone on the chondrogenesis of mesenchymal stromal cells: Influence of microenvironment, tissue origin and growth factor. Eur. Cells Mater..

[62] Weinstein R.S. (2012). Glucocorticoid-Induced Osteoporosis and Osteonecrosis. Endocrinol. Metab. Clin. N. Am..

