# Supplementary material for: Pharmacological Manipulation of Early Zebrafish Skeletal Development Shows an Important Role for Smad9 in Control of Skeletal Progenitor Populations
Source: Biomolecules. 2021 Feb 13;11(2):277. doi: 10.3390/biom11020277 (PMC7918065; doi:10.3390/biom11020277)
Supplement: Supplementary file 1 [file biomolecules-11-00277-s001.zip › Supplemental figures .pdf]

Pharmacological Manipulation of Early Zebrafish Skeletal Development Shows an Important Role for Smad9 in Control of Skeletal Progenitor Populations

Supplementary Figures

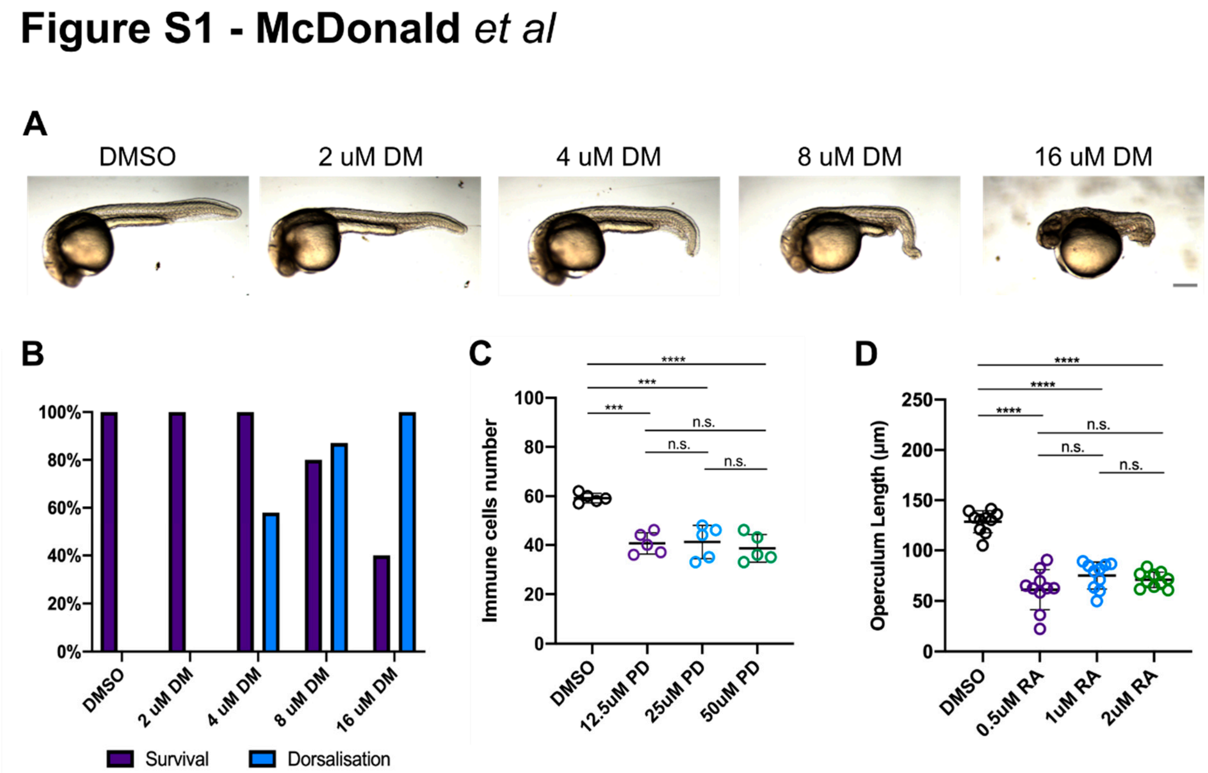

Figure S1: Dose response of pharmacological treatments.
A) Lateral view of larvae at 24 hours post-fertilisation (hpf) treated with DMSO and DM at a range of concentrations for 20 h. **B)** Quantification of survival and dorsalisation rate after DM treatment. Increasing the concentration of DM increased the dorsalisation of the tail and reduced the survival of larvae. **C)** Quantification of the number of immune cells in 5 dpf larvae treated with DMSO and PD at a range of concentrations for 48 h. Treating larvae with PD decreased the number of immune cells but there was no additional effect in immune cell reduction when increasing the concentration nor were there any morphological defects observed. **D)** Quantification of the length of the operculum in 5 dpf larvae treated with DMSO and RA at a range of concentrations for 48 h. Treating larvae with RA decreased the length of the operculum but there was no effect when increasing the concentration. Note, we observed adverse effects on the larval body axis (40%) using 2 µM RA. Scale bar is 200 µm.

Figure S2 - McDonald *et al*

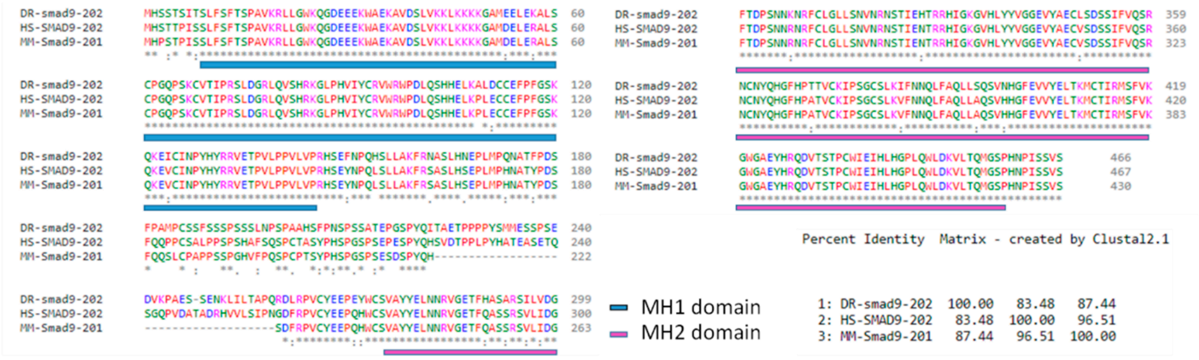

Figure S2: Smad9 protein is highly conserved between zebrafish and land mammals.
Clustal Omega protein sequence alignment of human (HS), mouse (MM), and zebrafish (DR) Smad9. Asterisk (*) shows residue homology in all three species, colon (:) indicates conserved residue charge with similar side chain properties and a stop (.) means a conserved residue charge. Full color coding and biochemical properties can be found on the Clustal Omega website (https://www.ebi.ac.uk/Tools/msa/clustalo/). The region of the MH1 and MH2 domains are indicated with a colored bar below the sequence. The linker region is in between the MH1 and MH2 domains.

Figure S3 - McDonald *et al*

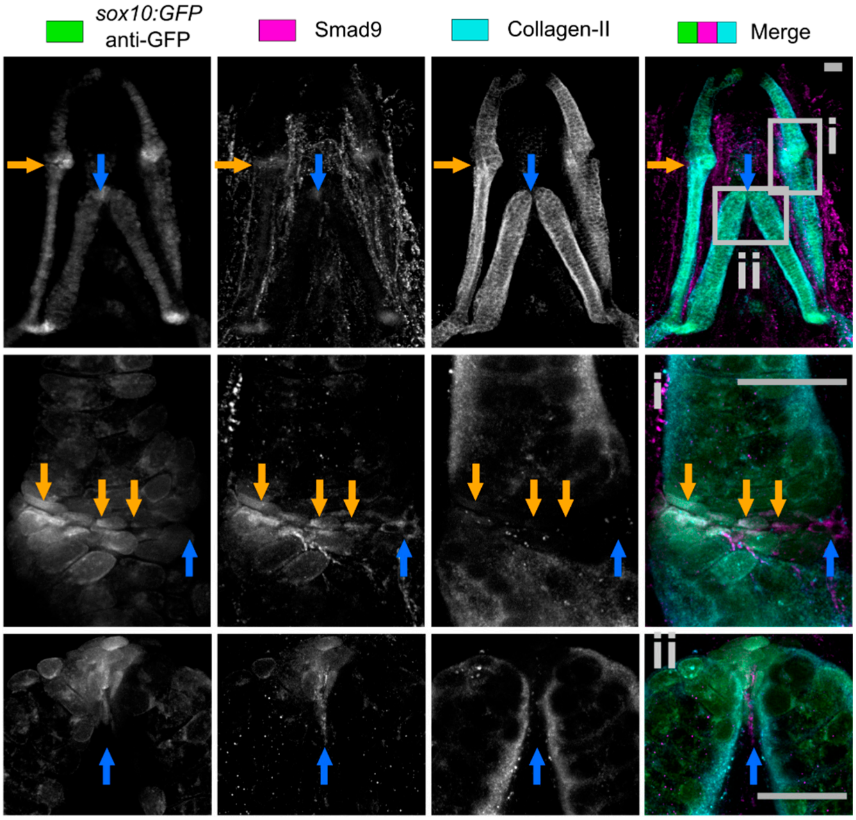

Figure S3: Smad9 is co-expressed with *sox10* at joint sites.
Ventral view of the jaw at 5 dpf labelled with anti-GFP (of *sox10:GFP*), Smad9 and type II Collagen, showing overlap of bright *sox10:GFP* expression with Smad9 in the PQ joint space (inset i, orange arrows), a small population of Smad9 expressing cells do not co-express *sox10:GFP* (inset i, blue arrow) indicating that Smad9 is downregulated in cells positive for type II Collagen. A similar observation was seen at the ceratohyal symphysis (inset ii, blue arrow) Smad9 expression partially overlaps GFP, but a small pocket of expression is seen in *sox10*- cells (blue arrow in ii). Scale bar 50 µm.

Figure S4 - McDonald *et al*

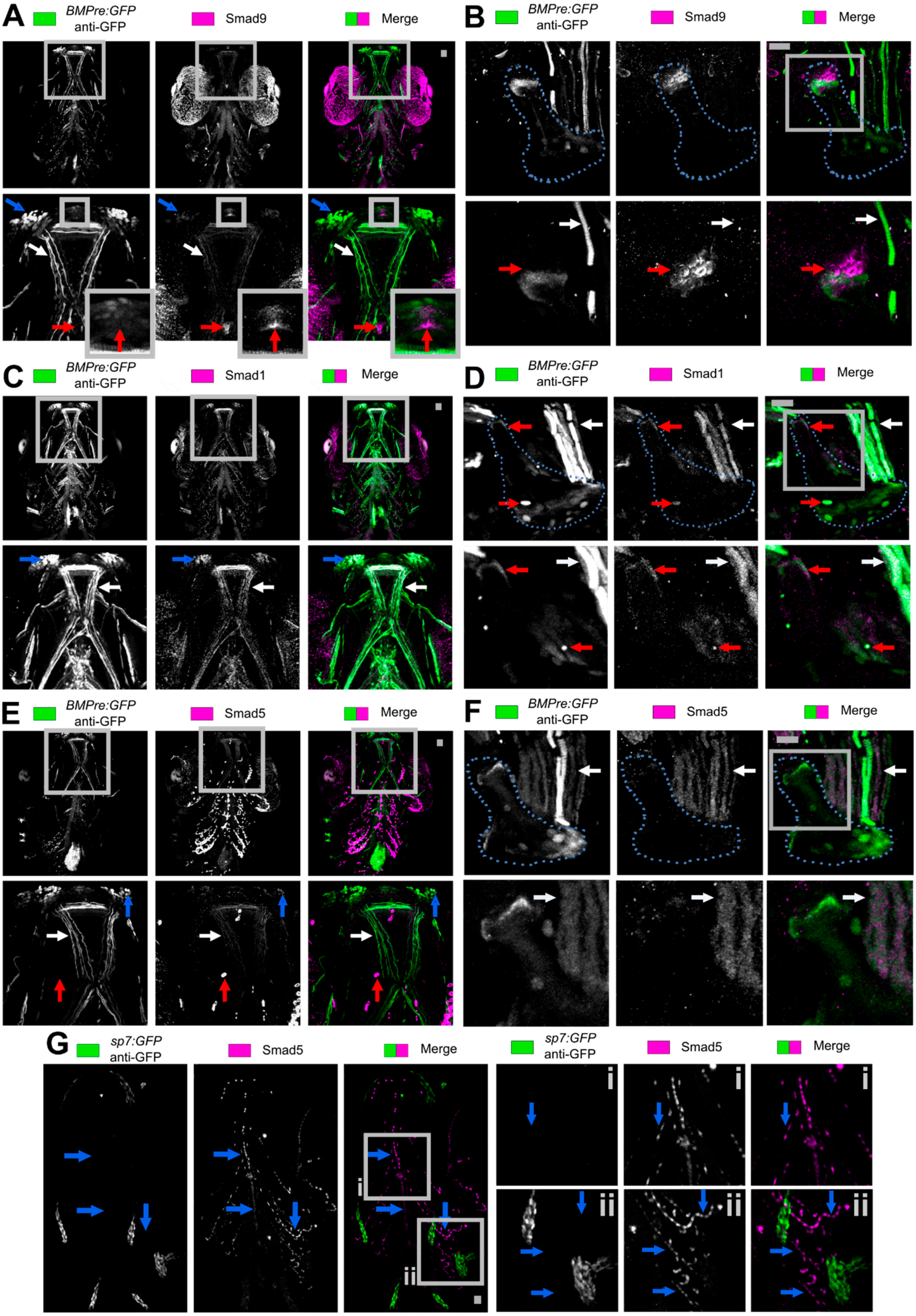

Figure S4: Smad9 expression does not overlap expression of BMP responsive cells, Smad1 or Smad5.

A) Ventral view of Smad9 and *BMPre:GFP* expression. Smad9 largely does not overlap *BMPre:GFP* at the MC symphysis (inset, red arrow) or ceratohyal symphysis (red arrow main image). Limited co-expression of Smad9 with *BMPre:GFP* expression in seen the jaw musculature (white arrow) and the olfactory placode (blue arrow). **B)** Lateral view of the operculum labelled for *BMPre:GFP* and Smad9. Smad9 expression is observed at the dorsal tip of the operculum (inset, red arrow) whilst GFP expression is weakly observed on the surface of the operculum. No overlap is observed in the muscle fibres which strongly expressed *BMPre:GFP* but not Smad9 (inset, white arrow). **C)** Ventral views of Smad1 and *BMPre:GFP*. Smad1 expression overlapped that of *BMPre:GFP* expression in muscle (inset, white arrow) and olfactory placode (inset, blue arrow). **D)** Lateral views of the operculum labelled for Smad1 and *BMPre:GFP*. Smad1 expression overlaps with *BMPre:GFP* expression in cells on the surface of the operculum (red arrows) and in surrounding muscle (white arrow). **E)** Ventral views of Smad5 and *BMPre:GFP* expression. Smad5 is weakly co-expressed with *BMPre:GFP* in skeletal muscle (inset, white arrow) (and to a lesser extent in cardiac muscle) and in the olfactory placode (inset, blue arrow). Smad5 also strongly expressed in a subset of vascular endothelial cells (inset, red arrow). **F)** Lateral views of Smad5 and *BMPre:GFP* expression. Smad5 is weakly co-expressed with *BMPre:GFP* in muscle adjacent to the operculum (white arrow) but is absent from the operculum. **G)** Expression of Smad5 and the osteoblast marker *sp7:GFP*. Smad5 not expressed in or around osteoblasts (inset ii, blue arrow) but is strongly expressed in a sub-population of vascular endothelial cells (inset i, blue arrow). All larvae 7 dpf, scale bar represents 20 μm.

Figure S5 - McDonald *et al*

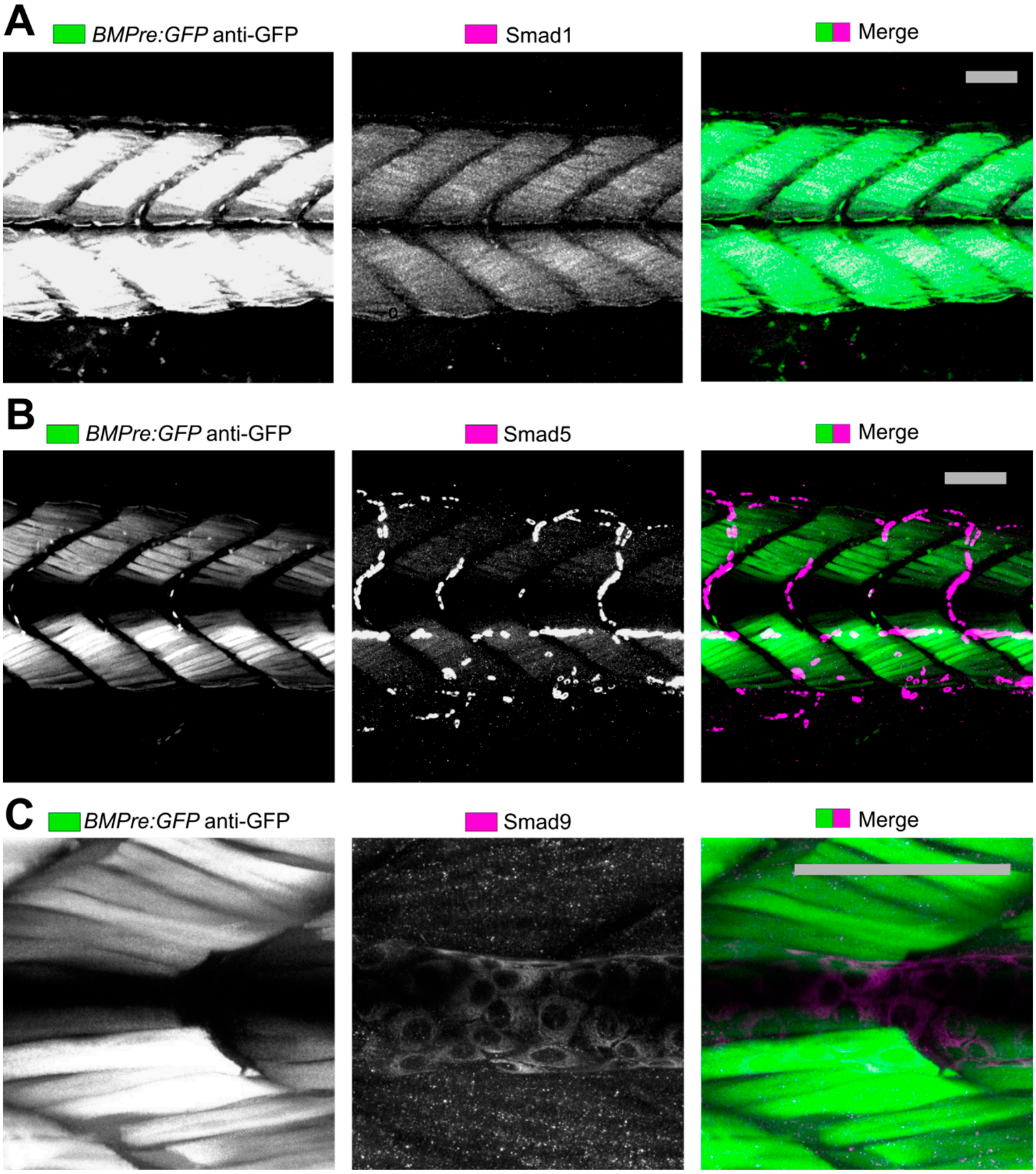

Figure S5: Smad9 is expressed in the notochord whilst Smad1, Smad5 and the *BMPre:GFP* transgene are not.

A) Smad1 expression overlapped *BMPre:GFP* expression in the trunk skeletal muscle. **B)** Smad5 is expressed in vascular endothelial cells of the inter-somitic vessels, and is weakly expressed in skeletal muscle where it overlaps *BMPre:GFP*. **C)** Smad9 is expressed strongly in notochord sheath cells, where no *BMPre:GFP* expression is seen. All individuals *BMPre:GFP* transgenic at 7 dpf. All panels show lateral views of the trunk at 7dpf. Scale bar is 20 μm.

Figure S6 - McDonald *et al*

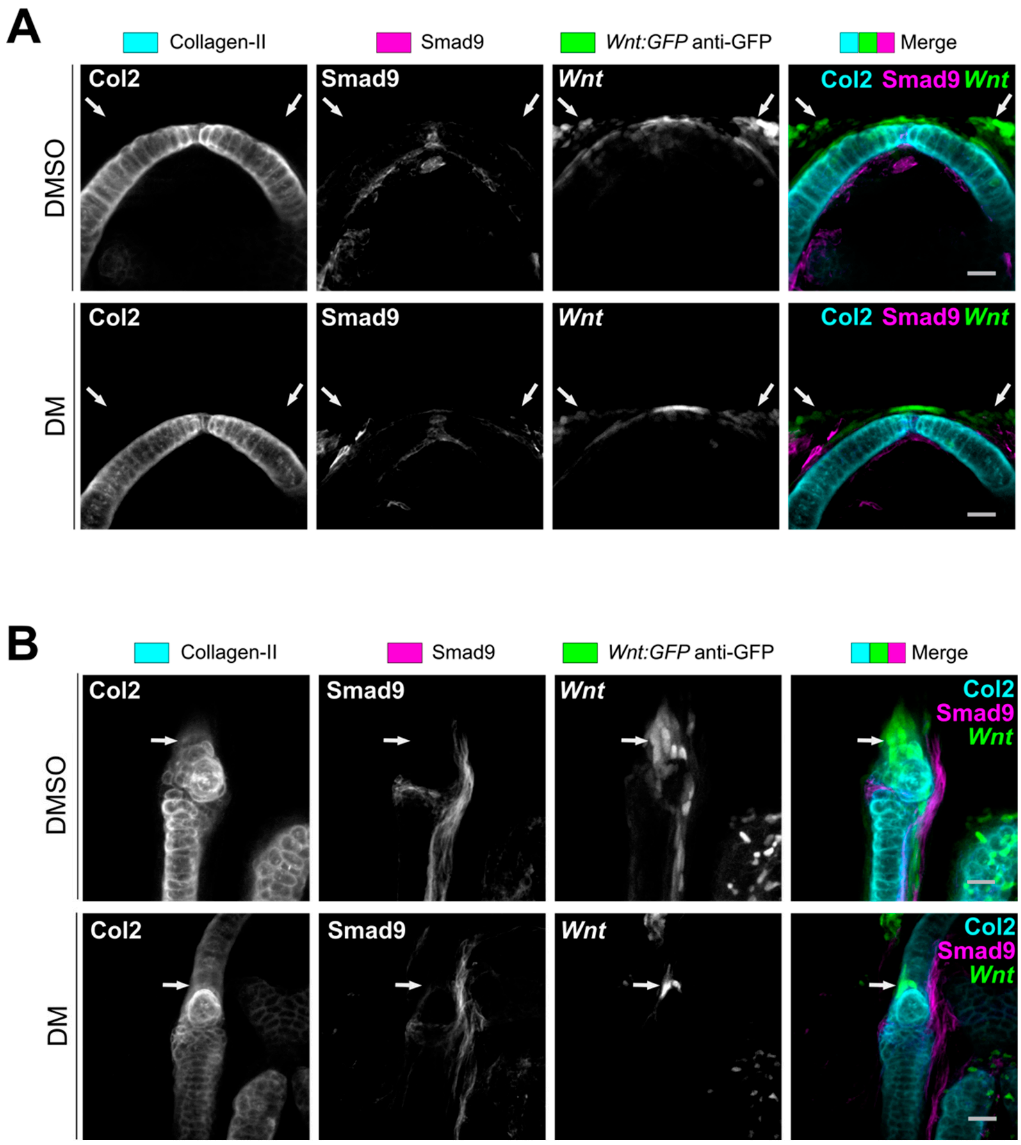

Figure S6: Pharmacological manipulation effects Wnt signalling in larvae jaws.
A) Ventral views of MC symphysis at 5 dpf labelled for type II Collagen, Smad9 and canonical *Wnt:GFP* reporter (Anti-GFP) in control and DM treated larvae. DM treatment decreased *Wnt* expression (white arrows). **B)** Ventral views of the MC-PQ synovial joint at 5 dpf labelled for type II Collagen, Smad9 and canonical *Wnt:GFP* reporter (Anti-GFP) in control and DM treated larvae. DM reduced *Wnt* expression at the joint site (white arrows). No overlap of Smad9 expression with *Wnt* in the joint spaces. Scale bar is 20 μm.

Figure S7 - McDonald *et al*

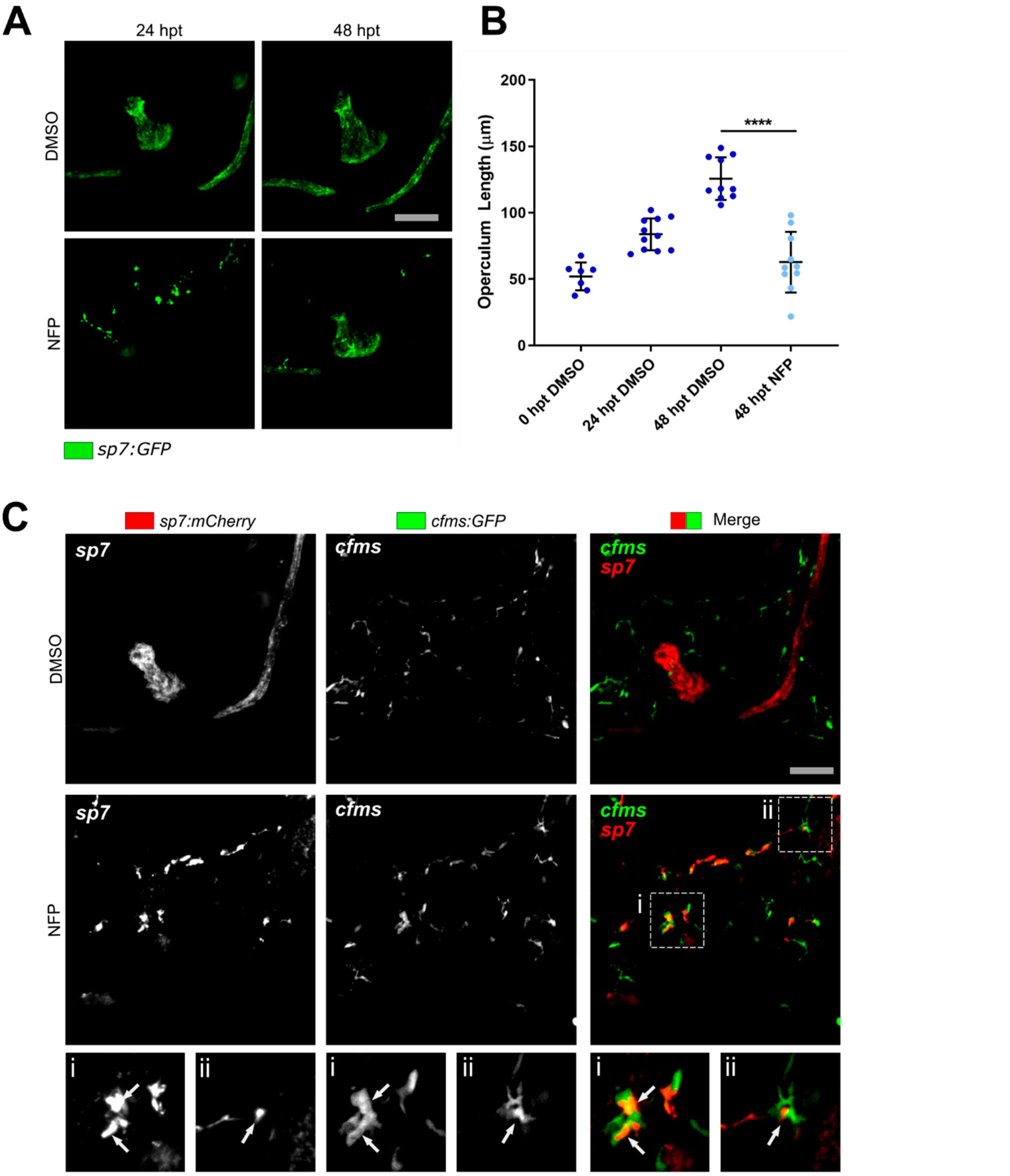

Figure S7: Chemical ablation of osteoblasts induces rapid macrophage clearance of apoptotic cells and subsequent regeneration of the operculum resident osteoblasts.

A) Lateral view of operculum at 24 hpt (4 dpf) and 48 hpt (5.25 dpf) when treated with DMSO and NFP for 6 hours at 3 dpf. The operculum is fully ablated at 24 hpt, osteoblast differentiation and operculum regeneration is observed by 48 hpt. **B)** Quantification of operculum length prior to (0 hpt) and after ablation (48 hpt) shows the operculum regenerates following the ontogenetic pattern. N ≥ 6. **C)** Lateral view of operculum at 24 hpt (4 dpf) showing *cfms* positive macrophages (*cfms:GFP*) engulfing ablated osteoblasts (inset i and ii, white arrow).

Movie S1: Rapid clearance of *sp7*+ cells by macrophages after chemical ablation.
Lightsheet time-lapse video of macrophages (green) engulfing the ablated *sp7:mCherry-NTR* osteoblasts (white) in the operculum during 6 hours NFP treatment at 3 dpf. Note, dorsal end of cleithrum could be seen at bottom right of the video. 15 mins per frame.
